# Cohort Profile: Scotland’s Substance Use and Health Intelligence Linked Dataset (SHIeLD)

**DOI:** 10.1093/ije/dyag140

**Published:** 2026-08-11

**Authors:** Lee Barnsdale, Alan Yeung, Megan Glancy, Jaroslaw Lang, Andreas Markoulidakis, Matthew Hickman, Hayley E Jones, Elinor Curnow, Kirsten M A Trayner, Rosalyn Fraser, Gordon Hunt, Femke de Wit, Andrew McAuley, Sharon Hutchinson, Tara Shivaji

**Affiliations:** Public Health Scotland, Edinburgh, United Kingdom; Public Health Scotland, Edinburgh, United Kingdom; School of Health and Life Sciences, Glasgow Caledonian University, Glasgow, United Kingdom; Public Health Scotland, Edinburgh, United Kingdom; School of Health and Life Sciences, Glasgow Caledonian University, Glasgow, United Kingdom; Public Health Scotland, Edinburgh, United Kingdom; Public Health Scotland, Edinburgh, United Kingdom; Population Health Sciences, Bristol Medical School, University of Bristol, Bristol, United Kingdom; School of Health and Life Sciences, Glasgow Caledonian University, Glasgow, United Kingdom; Population Health Sciences, Bristol Medical School, University of Bristol, Bristol, United Kingdom; National Drug and Alcohol Research Centre, University of New South Wales, Sydney, Australia; Population Health Sciences, Bristol Medical School, University of Bristol, Bristol, United Kingdom; Population Health Sciences, Bristol Medical School, University of Bristol, Bristol, United Kingdom; Public Health Scotland, Edinburgh, United Kingdom; School of Health and Life Sciences, Glasgow Caledonian University, Glasgow, United Kingdom; Public Health Scotland, Edinburgh, United Kingdom; School of Health and Life Sciences, Glasgow Caledonian University, Glasgow, United Kingdom; Public Health Scotland, Edinburgh, United Kingdom; Public Health Scotland, Edinburgh, United Kingdom; Public Health Scotland, Edinburgh, United Kingdom; School of Health and Life Sciences, Glasgow Caledonian University, Glasgow, United Kingdom; Public Health Scotland, Edinburgh, United Kingdom; School of Health and Life Sciences, Glasgow Caledonian University, Glasgow, United Kingdom; Public Health Scotland, Edinburgh, United Kingdom

**Keywords:** opioids, opioid agonist therapy, substance use, drug-related deaths, public health

Key Features

- Scotland’s Substance Use and Health Intelligence Linked Dataset (SHIeLD) was established to address public health policy and research aims (prevalence, morbidity and mortality, treatment effectiveness) in relation to Scotland’s drug-related-death public health emergency.
- Inclusion in SHIeLD is based on records (from 1 April 2009 onwards) from multiple national administrative databases containing information on treatments or harms related to opioids and other problematic drug use.
- Data are linked across administrative databases via a unique identifier (the Community Health Index number), which means that the dataset is extendable and able to incorporate other relevant information, e.g. on prison custody, hepatitis C, and HIV testing and treatment.
- Through SHIeLD, a subcohort of all individuals in receipt of opioid agonist therapy in Scotland has been established for between the years 2011 and 2022: *n* = 49 392 individuals, 67% male and with a median age of 36 years at cohort entry.
- Further information is available upon request from phs.shield@phs.scot. Cohort data are available for research subject to Public Benefit and Privacy Panel permission (contact phs.edris@phs.scot for further details).

## Why was the cohort set up?

The Substance Use and Health Intelligence Linked Dataset (SHIeLD) [1] is a national, population-based, retrospective, linked administrative open cohort of people with problematic drug use in Scotland, with an initial focus on those prescribed opioid agonist therapy (OAT). It is based on multiple administrative databases recording drug-related health harms, treatments, and other exposures held by Public Health Scotland (PHS). SHIeLD was established in 2019 to address three main public health policy and research aims:

- To estimate the size and composition of Scotland’s population of people dependent on opioids.
- To establish trends in morbidity, drug-related mortality, and other causes of death among people who use drugs.
- To assess the impact of OAT and other interventions on morbidity and mortality.

In the context of rapidly increasing numbers and globally high rates of drug-related deaths (DRDs), most commonly due to opioid overdose, addressing the harms associated with problematic drug use is a public health emergency for Scotland [2]. From 2014 onwards, the number of DRDs registered in Scotland has increased year on year, reaching a peak of 1339 in 2020 (245 DRDs per million population), on a par with increases seen in the USA (277 DRDs per million population in 2020). While the number of DRDs has since plateaued (1017 in 2024), Scotland’s DRD rate remains above that of other UK and European countries, and is among the highest in the world [3, 4].

Alongside policy initiatives that have formed Scotland’s response to this public health emergency since the late 2010s, it was recognized that a better understanding of the problem through estimating the size of the population at risk, the uptake of OAT, and the factors associated with drug-related mortality and successful treatment was crucial for informing public health responses.

Additionally, during the COVID-19 pandemic, an adapted SHIeLD dataset was utilized to monitor and evaluate COVID-19 testing, infections, and vaccine uptake, and other impacts among people prescribed OAT [5–7].

## Who is in the cohort?

**Figure 1:**
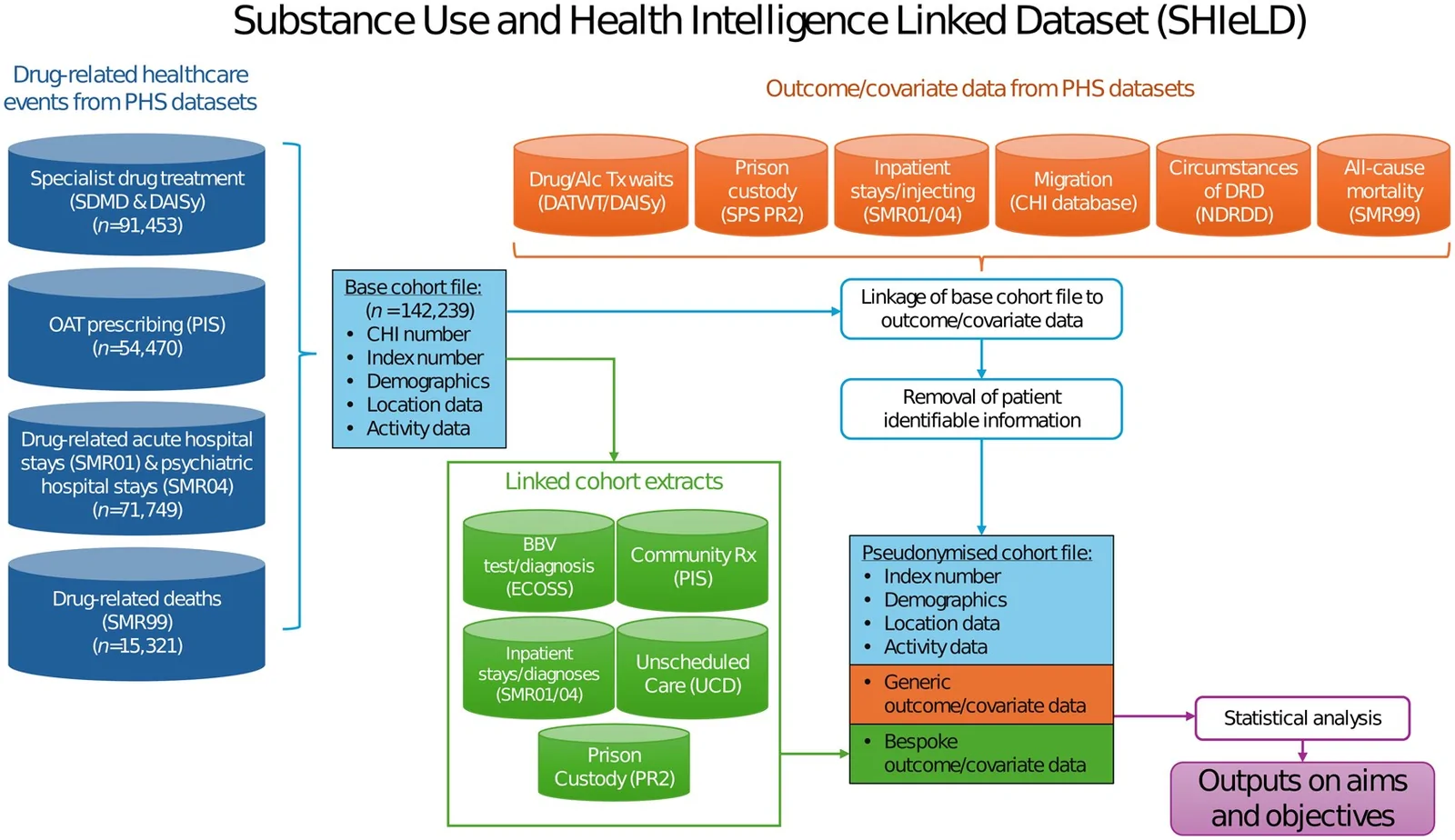
Overview of the record-linkage process for creating the SHIeLD data resource. The number of individuals identified from each drug-related-event database and the number of unique individuals identified based on data from 1 January 2009 to 31 March 2024 are shown within the boxes on the left-hand side.

People are included in SHIeLD on the basis of records from several national administrative databases that hold information on treatments or harms related to problematic drug use. Specifically, people with one or more drug-related events as recorded on the following PHS databases (referred to as “identification databases,” i.e. databases used to identify individuals with problematic drug use) from 1 April 2009 onwards are included (Table 1 and see Fig. 1 for a summary of the number of individuals included from different data sources and an overview of the data-linkage process):

**Table 1:** Databases used to identify drug-related events for inclusion in the SHIeLD data resource.

Database name	Description	Definition	Key variables	Start date for data extraction
Scottish Drug Misuse Database (SDMD)	National database describing people experiencing problematic drug use and seeking specialist treatment in Scotland, including in prisons	All episodes of care	Date of assessment NHS Board of residence Illicit drug use Injecting status Accommodation status Prison custody Age started using drugs Age of problematic drug use Discharge date Discharge type	April 2009
Drug and Alcohol Information System (DAISy)	National database describing people experiencing problematic drug or drug and alcohol use (codependency) and seeking specialist treatment in Scotland, including in prisons. DAISy replaced SDMD from April 2021.	All drug or codependency episodes of care		April 2021
Prescribing Information System (PIS)	Subset of data from Scottish community prescribing dataset, describing people to whom an OAT prescription was dispensed	Legacy British National Formulary (BNF) subsection 04.10.03, excluding lofexidine and naltrexone	BNF item code	April 2009
			Date item prescribed	
			Date item dispensed	
			Date of reimbursement for item	
			NHS Board of residence	
Scottish Morbidity Records 01 (SMR01)	Subset of data from Scottish general acute inpatient and day-case hospital admissions dataset, describing people who had a drug-related hospital stay	Hospital inpatient discharges relating to illicit drugs, defined by ICD10 codes F11, F12, F13, F14, F15, F16, F18, F19, T40.0, T40.1, T40.3, T40.5, T40.6, T40.7, T40.8, T40.9	Admission date Discharge date ICD-10 diagnosis code NHS Board of residence	April 2009
Scottish Morbidity Records 04 (SMR04)	Subset of data from Scottish psychiatric inpatient and day-case hospital admissions dataset, describing people who had a drug-related hospital stay			
Scottish Morbidity Records 99 (SMR99)	Subset of data from National Records of Scotland (NRS) death-registration dataset, restricted to confirmed DRDs only	DRDs as per UK Drug Strategy definition	Date of death	January 2009
			Date of registration of death	
			Location of death	
			NHS Board of residence	
			Cause(s) of death Drug(s) implicated in death Drug(s) present at postmortem	

**Table 2:** Demographic characteristics, deaths, and exposure to OAT in the OAT subcohort identified through SHIeLD, 1 January 2011 to 31 December 2022.

	OAT cohort [*n* (% in group)]
**Total**	**49 392 (100.0)**
**Sex**	
Male	33 289 (67.4)
Female	16 103 (32.6)
**Age group (at cohort entry) (years)**	
16–34	22 780 (46.1)
35–44	18 821 (38.1)
45–69	7791 (15.8)
**NHS Board (at last known address)**	
Ayrshire and Arran	4166 (8.4)
Fife	2921 (5.9)
Grampian	4781 (9.7)
Greater Glasgow and Clyde	15 164 (30.7)
Lanarkshire	5038 (10.2)
Lothian	7656 (15.5%)
Tayside	4042 (8.2)
Rest of Scotland	5410 (11.0)
Missing	214 (0.4)
**Deprivation (SIMD quintile at last known address)**	
SIMD 1 (most deprived)	25 690 (52.0)
SIMD 2	12 465 (25.2)
SIMD 3–5	11 040 (22.4)
Missing	197 (0.4)
**Year of entry to cohort (first observed prescription of OAT)**	
2011–13	33 413 (67.6)
2014–16	6499 (13.2)
2017–19	5164 (10.5)
2020–22	4199 (8.5)
**In receipt of OAT (at least once during time period)**	
2011–13	33 413 (67.6)
2014–16	34 435 (69.7)
2017–19	34 774 (70.4)
2020–22	33 586 (68.0)
**Ever prescribed OAT**	
Methadone only	30 529 (61.8)
Buprenorphine only	5637 (11.4)
Methadone and buprenorphine	13 226 (26.8)
**Person-years of observation**	
Total	365 219
In OAT	268 030
Out of OAT	97 189
**Died during observation**	
Any cause	8899 (18.0)
Of which, drug-related death	5231 (10.6)

- Prescribing Information System (PIS)—identifies people in receipt of community-based OAT prescriptions for medications listed in legacy British National Formulary (BNF) subsection 04.10.03, including methadone hydrochloride, buprenorphine, buprenorphine–naloxone, and long-acting buprenorphine. Prescription medicines are available free of charge in Scotland.
- The Scottish Drug Misuse Database (SDMD) and the Drug and Alcohol Information System (DAISy) identify people using specialist drug treatment services.
- Scottish Morbidity Records (SMR) acute (SMR01) and psychiatric (SMR04) hospital discharges identify people admitted to hospital in relation to their drug use.
- Scottish Morbidity Records death registrations (SMR99) are a subset of the death-registration database collated by the National Records of Scotland (NRS), identifying people who experience a DRD.

Based on data from the financial years 2009/10 to 2023/24, ∼142 000 people were included in SHIeLD, with ∼6500 being added each year. While the scope of the SHIeLD cohort (relating to all problematic drug use) is intentionally wide to enhance public health surveillance and facilitate research across the breadth of drug-related health data that PHS holds, initial analyses have focused on people with an opioid dependence, as they account for the majority of DRDs over the last decade in Scotland. Thus, the first subcohort established by using SHIeLD focused on people prescribed OAT, as recorded in PIS.

There were 49 392 people included in the OAT subcohort between 2011 and 2022 (Table 2). One-third (32.6%) were female and the median age at baseline (initial OAT prescription) was 36 years. Over half (52.0%) lived in areas of the most deprived quintile [measured by using the Scottish Index of Multiple Deprivation (SIMD)]. While most (61.8%) had been treated with methadone only, a considerable minority (38.2%) had experience of buprenorphine prescribing. Up to the end of calendar year 2022, there were 8899 (18.0%) deaths among the OAT subcohort, of which 5231 (58.8% of observed deaths) were drug-related (representing 47.6% of all DRD registrations in Scotland during this period).

## How often have they been followed up?

SHIeLD is an open, longitudinal retrospective cohort. Individuals enter the cohort at the date of their first qualifying drug-related record (since April 2009) and are identified by using the Community Health Index (CHI) number (the unique patient identifier used across healthcare services in Scotland).

Extracts from identification datasets are updated annually to capture newly eligible individuals (Table 1). All cohort members are then followed longitudinally through deterministic linkage to national administrative datasets containing person-level information on prescribing, hospital admissions, laboratory testing, and mortality (see Supplementary Table 1 for further details on linked databases). These linked data extracts are also updated annually.

Follow-up time accrues from cohort entry until death, migration out of Scotland, or the end of available data.

## What has been measured?

Current patient demographic data are obtained by linkage to the CHI database, which contains information on current place of residence, SIMD, and migration out of Scotland. Linkage to SMR databases, PIS, and Electronic Communication of Surveillance Scotland (ECOSS) data allows clinical information to be derived from hospital admissions, prescriptions, and laboratory test results for pathogens, respectively. For example, blood-borne virus infection status can be obtained from ECOSS data and specific comorbidities can be identified by searching for hospital admissions with specific ICD-10 diagnosis codes or prescriptions with specific BNF codes [8, 9]. These can also be used to generate a Charlson Comorbidity Index [10]. Details of the variables available in these databases are provided in Supplementary Table 1.

The linked dataset also includes data on social factors related to problematic drug use. SDMD and DAISy provide self-reported data on illicit drug use, injecting status, homelessness, and experience of prison custody. Similar data, sourced from local health and justice records obtained as part of National Health Service (NHS) Board drug-related-death reviews, are also available (for drug-death decedents only) from the National Drug Related Deaths Database (NDRDD). Work is underway to link administrative information on prison-custody episodes to people in the SHIeLD cohort by using data from the Scottish Prison Service’s prisoner records system (PR2).

SHIeLD includes death-registration data (from SMR99) for all relevant cohort members, facilitating analyses of all-cause mortality and cause-specific mortality via International Classification of Disease version 10 (ICD-10) codes. DRDs are identified in NRS mortality records (National Records of Scotland, 2023) and include all deaths for which the underlying cause of death was attributed to substance use (F11–F16, F18, F19) or any contributing cause was attributed to drug-related accidental poisoning (X40–X44), intentional poisoning (X60–X64), assault (X85), or poisoning of undetermined intent (Y10–Y14). Analysis of specific substances involved in confirmed DRDs is possible through the linkage of additional toxicology information supplied by NRS.

For the OAT subcohort, prescription-level PIS data permit the detailed characterization of OAT exposure. Individual prescriptions can be used to construct time-updated treatment episodes, enabling classification of periods on and off treatment and examination of risk during specific stages of therapy, including initiation and cessation. Prescription data also allow the assessment of co-prescribing of other controlled drugs (e.g. benzodiazepines, z-hypnotics, and gabapentinoids). These features support analyses of treatment effectiveness, high-risk periods, and medication-related risk.

## What has it found?

### Opioid prevalence

A key output from the OAT subcohort established through SHIeLD is the estimation of the prevalence of opioid dependence via a novel Bayesian statistical modeling approach: “multi-parameter estimation of prevalence” (MPEP) [11, 12]. The prevalence of opioid dependence was high compared with those of the rest of the UK and many other countries. However, since 2014/15, the prevalence has not increased and may have fallen slightly. In 2022/23, we estimated that the number of people with opioid dependence was 43 400 (95% credible interval: 41 900 to 45 100) in Scotland, equivalent to 1.23% (95% credible interval: 1.19% to 1.28%) of those aged 15–64 years [12, 13]. New estimates covering the period to 2023/24 will be published in 2026.

### Morbidity, mortality, and the effectiveness of interventions

The OAT subcohort has generated critical evidence on trends in the risk of DRD. We showed that DRD mortality among this population had more than tripled from 6.36 per 1000 person-years in 2011–12 to 21.45 per 1000 person-years in 2019–20 [14].

To examine the protective effect of OAT in Scotland, community prescribing data from SHIeLD has enabled the characterization of OAT treatment episodes. Although mortality risk increased in patients on as well as off OAT, the average mortality risk among people on OAT was 70% lower than that among people out of OAT [14]. These trends were extended to cover the pandemic period, showing that the DRD mortality risk did not increase among those in the OAT subcohort during the COVID-19 pandemic in Scotland and that the protective effect of OAT was maintained [15].

**Figure 2:**
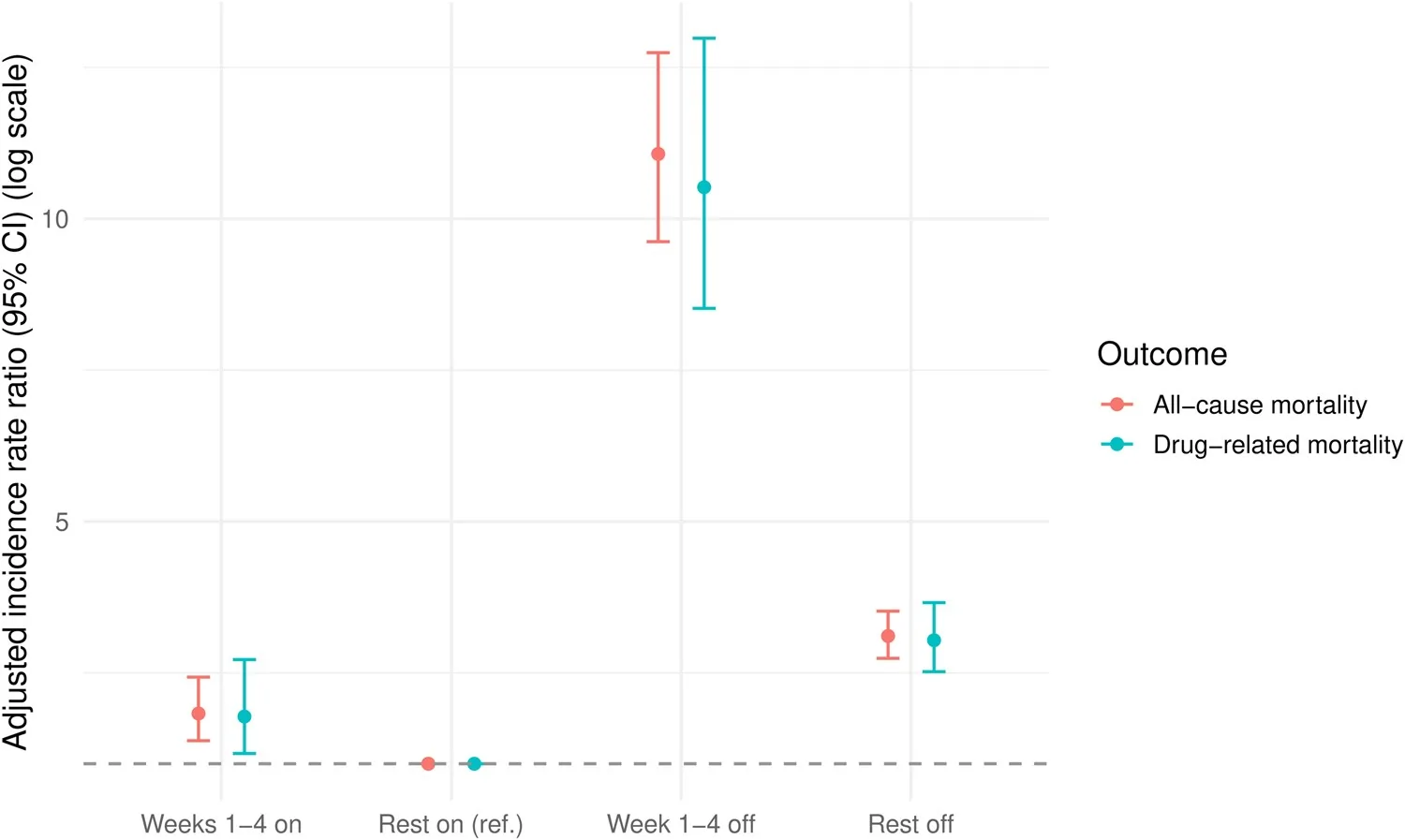
Analysis of association between OAT treatment period (reference group, 4+ weeks on OAT) and all-cause and drug-related mortality, 2011–22 (*n* = 49 392 individuals). Adjusted incidence rate ratios and 95% confidence intervals are presented. Missing prescription dates were addressed by using MI to define time-updated OAT exposure (1–4 weeks on, ≥4 weeks on, 1–4 weeks off, ≥4 weeks off). Estimates were obtained by using pooled quasi-Poisson regression with person-time as an offset, adjusted for medication type, age, sex, NHS Board, deprivation quintile, and previous non-fatal overdose.

Exact OAT start and finish dates are missing from ∼56% of prescription records, though the pharmacy reimbursement date (which occurs at the end of the month after the prescription duration finishes) is available for all prescriptions. The first set of analyses comparing the risk of being on versus out of OAT relied on an algorithm based on this reimbursement date. These analyses were repeated with OAT episode definitions refined by using multiple imputation (MI) to address missing prescription dates. The results from the MI analysis were consistent with those of previous analyses (Supplementary Table 2). In addition, analyses were extended to examine the risk during specific treatment stages (Fig. 2). The mortality risk was markedly elevated during the first 4 weeks following treatment cessation compared with sustained treatment, consistently with known periods of heightened vulnerability [16].

Prescription-level data have also been used to examine co-prescribing. Exposure to benzodiazepines, z-hypnotics, and gabapentinoids among OAT recipients was associated with increased risk of DRD [17].

Beyond the OAT subcohort, SHIeLD has been used to examine outcomes among ∼6500 patients following drug-related hospitalization. People with opioid-related discharges were 1.8 times more likely than people with non-opioid drug-related discharges to die of drug-related causes and 1.5 times more likely to die of any cause. The 10-year all-cause survival rate was lower among people with opioid-related discharges compared with those with non-opioid-drug-related discharges in all age groups up to 45 years [18].

The Scottish Suicide Information Database (ScotSID) uses SHIeLD data to describe the prevalence of problematic drug use among those who died by suicide. Contact with a specialist drug treatment service was observed in 1.2% of suicides in the period from 2011 to 2024—six times more commonly than in the general population (0.2%). OAT was also associated with a lower risk of suicide in people who were opioid-dependent [19].

### COVID-19 risk and vaccination

The OAT subcohort was used to examine factors associated with COVID-19 testing and diagnosis as well as vaccine uptake and the risk of severe COVID-19 disease (admission to critical care within 28 days of testing polymerase chain reaction (PCR)-positive or death with COVID-19 as the underlying cause). Those with a recent OAT prescription (<2 months ago) had lower odds of testing positive for COVID-19 compared with past exposure to OAT (adjusted odds ratio 0.63, 95% confidence interval (CI) 0.57 to 0.69) [5]. Vaccine uptake in the OAT subcohort lagged behind a control group from the general population, matched by sociodemographic factors. The gap in vaccine coverage widened with each successive dose, reaching a 26% gap by Dose 3. This likely exacerbated the risk of severe COVID-19 disease among those prescribed OAT as the pandemic progressed [adjusted risk ratio (aRR) 3.78, 95% CI 2.30 to 6.20 in Wave 4: December 2021 to March 2022]. Those with past exposure to OAT had a higher risk of severe COVID-19 disease compared with those with recent OAT (aRR 1.74, 95% CI 1.11 to 2.71) [7].

## What are the main strengths and weaknesses?

SHIeLD is a national linked data resource with extensive coverage of drug-related contacts with healthcare services in Scotland. It is a key public health surveillance resource, facilitating research into policy themes associated with drug-related harms in Scotland. The duration of the cohort and the ability to link a range of data sources via a robust patient identifier (CHI) with high coverage mean that questions can be addressed with strong statistical power. SHIeLD therefore enables population-level analyses so as to understand the drivers of the ongoing public health crisis and to inform effective policy and service responses. A particular strength is that community prescribing data are available at the level of individual prescriptions, allowing time-dependent modeling of exposure to OAT and other medications. This facilitates detailed analyses of treatment episodes and transitions on and off therapy in relation to mortality and other outcomes.

There are, however, a number of limitations. First, the incomplete availability of CHIs in some administrative datasets may have led to under-ascertainment. Although CHI completeness exceeds 99% in hospital records (SMR01, 04, 99), it is lower in prescribing and specialist treatment datasets (SDMD/DAISy and PIS). For example, the CHI availability for OAT prescriptions in PIS increased from 56% in 2009/10 to 85% in 2023/24 [20], with regional variation. Records without CHIs may not be successfully linked to supplementary datasets, potentially introducing selection bias if CHI availability differs systematically by patient/treatment characteristics.

Second, some information sources about problematic drug use are missing and/or variables from linked datasets are incomplete. For example, specialist drug treatment assessment data (SDMD/DAISy) had an estimated completeness of 67% of eligible treatment episodes in 2024/25 [21], resulting in missing information on illicit drug use, injecting status, and homelessness. While identification of the OAT subcohort via PIS data is robust, identification of people using non-opioid drugs may be less complete. Work is ongoing to improve DAISy assessment recording and address the underrepresentation of individuals not engaged with services. Data on drug-related urgent-care events from PHS’s Unscheduled Care Datamart (UCD) are being assessed for inclusion as an “identification dataset.” This will broaden the scope of the SHIeLD cohort, by identifying people who were treated for drug intoxication or overdose in an urgent-care setting but are not recorded in SHIeLD’s current identification datasets (which have tended to focus on opioid-related treatment and harms). The reliability of cohort identification for other drug types (e.g. cocaine) could also be enhanced if supplementary datasets specified illicit drug use more accurately or via the inclusion of other healthcare (e.g. primary care) or non-healthcare (e.g. police arrests) data.

Third, medication-dose data are limited to the total quantity dispensed over the duration of each prescription. This is problematic for medications such as OAT, for which prescription lengths may vary (over time and from person to person) and dispensing is often in installments (e.g. daily). “Dose instruction” data are currently unavailable due to the unintentional recording of sensitive information in these fields. This issue may be addressed in future through the application of Natural Language Processing [22, 23].

## Can I get hold of the data? Where can I find out more?

Requests to access SHIeLD data can be made by emailing phs.shield@phs.scot. Cohort data are available for research subject to Public Benefit and Privacy Panel permission (www.informationgovernance.scot.nhs.uk/pbpphsc/home/for-applicants). Contact phs.edris@phs.scot for further details.

## Ethics approval

Approval for the analysis of linked data at PHS was provided by the NHS Public Benefit and Privacy Panel for Health and Social Care (PBPP 1819–0235). A Data Protection Impact Assessment has also been approved by information governance at PHS (DP20210129).

## Supplementary Material

dyag140_Supplementary_Data
