# Supplementary material for: Cohort Profile: Scotland’s Substance Use and Health Intelligence Linked Dataset (SHIeLD)
Source: Int J Epidemiol. 2026 Aug 11;55(4):dyag140. doi: 10.1093/ije/dyag140 (PMC13456961; doi:10.1093/ije/dyag140)
Supplement: Supplementary file 1 [file dyag140_supplementary_data.docx]

Supplementary Information to accompany *Cohort Profile: Scotland’s Substance Use and Health Intelligence Linked Dataset (SHIeLD)*.

**Supplementary Table S1** Linked databases and details of key outcome/covariate variables included in the Substance Use and Health Intelligence Linked Dataset (SHIeLD) data resource.

| **Database Name** | **Database Description** | **Key Variables** | **Further Information** |
| --- | --- | --- | --- |
| Community Health Index (CHI) Database | Patient details for individuals registered with healthcare services in Scotland. This includes information such as a patient’s most recent known place of residence (from which deprivation information can be derived) and migration out of Scotland. | NHS board of residence | NHS board of residence derived from postcode of residence provided at most recent interaction with healthcare services. |
|  |  | SIMD quintile | Scottish Index of Multiple Deprivation (SIMD) quintile derived from postcode of residence. |
|  |  | Transfer out | Record of migration out of Scotland |
|  |  | Date transferred out | Date of migration out of Scotland |
| Drug and Alcohol Information System Waiting Times (DAISy_WT) | Data on referrals for specialist drug, alcohol or co-dependency treatments in Scotland (from 2021 onwards) | Referral date  Referral type  Date treatment started  Treatment type  Discharge date  Discharge type |  |
| Drug and Alcohol Treatment Waiting Times database (DATWT) | Data on referrals for specialist drug or alcohol treatment in Scotland (up to 2021) |  |  |
| Electronic Communication of Surveillance in Scotland (ECOSS) | Laboratory testing results for hepatitis B virus, hepatitis C virus and HIV. | Date specimen collected  HCV antibody test result  HCV antigen test result  HCV PCR test result  HIV antibody test result  HIV viral load test result |  |
| Homeless Registrations (SOURCE) | Details of homelessness registrations made with Scottish local authorities |  |  |
| National Drug Related Death Database (NDRDD) | Details of the health and social circumstances of people who died of DRD in Scotland. | Known drug user? |  |
|  |  | Known injecting drug user? |  |
|  |  | In contact with drug treatment services? |  |
|  |  | Living where? | Accommodation status prior to death |
|  |  | Drugs present at post-mortem |  |
|  |  | Drugs implicated in death | Substances considered implicated in, or potentially contributing to death, following pathology examination |
| Prescribing Information System (PIS) | Details of all prescriptions dispensed in the community | BNF item code | British National Formulary code describing prescribed item |
|  |  | Date item prescribed | Available for electronic prescriptions only (otherwise, defaults to date of reimbursement) |
|  |  | Date item dispensed |  |
|  |  | Date of reimbursement for item | Date of reimbursement for prescriptions which is set as the end of a month for a prescribed regimen. |
|  |  | NHS board of residence | NHS Board of residence for a patient at the time an item is prescribed. |
| Scottish Morbidity Records 01 (SMR01) | All-cause general acute inpatient and day-case hospital admissions | Admission date |  |
|  |  | Discharge date |  |
|  |  | ICD-10 diagnosis code | Conditions treated during hospital admission. |
| Scottish Morbidity Records 04 (SMR04) | All-cause psychiatric inpatient and day-case hospital admissions |  |  |
| Scottish Morbidity Records 99 (SMR99) | All-cause mortality data from National Records of Scotland (NRS). | Date of death |  |
|  |  | Date of registration of death | Date death registered with NRS – used to assign deaths to specific years |
|  |  | Location of death |  |
|  |  | NHS board of residence | NHS board of residence at time of death |
|  |  | Cause(s) of death | ICD-10 diagnostic codes related to main (underlying) and other causes of death, as determined by doctor/pathologist |
| Scottish Prison Service Custodial Records (SPS PR2) | Data on receptions into, and liberations from prison custody in Scotland. |  |  |

**Supplementary Table S2** Adjusted and unadjusted analysis of association between opioid agonist therapy (OAT) and treatment period and all-cause and drug-related mortality, 2011 to 2022 (*n* = 49,392 individuals). Previously published estimates from 2011–2020 (McAuley et al., 2023) and 2011–2022 (Glancy et al., 2025) are shown for comparison.

|  | **Category** | **PY^a^** | **Deaths^a^** | **CMR^a^** | **IRR (95% CI)** | **P value** | **aIRR^b^ (95% CI)** | **P value** |
| --- | --- | --- | --- | --- | --- | --- | --- | --- |
| ***Drug-related mortality*** | | | | | | | | |
| Model 1 | Overall on (ref.) | 268,030 | 2,219 | 8.28 | 1 (ref.) |  | 1 (ref.) |  |
|  | Overall off | 97,189 | 3,012 | 31.20 | 3.77 (3.09, 4.60) | <0.001 | 4.11 (3.47, 4.87) | <0.001 |
| ***McAuley et al. (2023)*** | *Overall on (ref.)* | *223,973* | *1,832* | *8.18 (7.81, 8.56)* | *1 (ref.)* |  | *1 (ref.)* |  |
|  | *Overall off* | *80,069* | *2,244* | *28.03 (26.88, 29.20)* | *3.43 (2.79, 4.22)* | *<0.001* | *3.37 (1.74, 6.53)* | *<0.001* |
| ***Glancy et al. (2025)*** | *Overall on (ref.)* | *271,722* | *2,452* | *9.02 (8.67, 9.39)* | *1 (ref.)* |  | *1 (ref.)* |  |
|  | *Overall off* | *94,622* | *2,803* | *29.62 (28.54, 30.74)* | *3.28 (2.82, 3.83)* | *<0.001* | *3.47 (2.98, 4.05)* | *<0.001* |
|  |  |  |  |  |  |  |  |  |
| Model 2 | Weeks 1-4 on | 15,171 | 209 | 13.80 | 1.73 (1.06, 2.84) | 0.029 | 1.78 (1.17, 2.72) | 0.008 |
|  | Rest on (ref.) | 252,858 | 2,010 | 7.95 | 1 (ref.) |  | 1 (ref.) |  |
|  | Week 1-4 off | 14,892 | 1,220 | 81.90 | 10.3 (8.06, 13.16) | <0.001 | 10.52 (8.52, 12.98) | <0.001 |
|  | Rest off | 82,297 | 1,830 | 22.02 | 2.77 (2.23, 3.44) | <0.001 | 3.04 (2.52, 3.66) | <0.001 |
|  |  |  |  |  |  |  |  |  |
| ***All-cause mortality*** | | | | | | | | |
| Model 1 | Overall on (ref.) | 268,030 | 3,744 | 13.97 | 1 (ref.) |  | 1 (ref.) |  |
|  | Overall off | 97,189 | 5,155 | 53.04 | 3.80 (3.32, 4.35) | <0.001 | 4.25 (3.79, 4.77) | <0.001 |
| ***McAuley et al. (2023)*** | *Overall on (ref.)* | *223,973* | *2,974* | *13·28 (12·81, 13·76)* | *1 (ref.)* |  | *1 (ref.)* |  |
|  | *Overall off* | *80,069* | *3,973* | *49·62 (48·09, 51·18)* | *3·74 (3·19, 4·38)* | *<0·001* | *4·08 (3·49, 4·76)* | *<0·001* |
| ***Glancy et al. (2025)*** | *Overall on (ref.)* | *271,722* | *3,974* | *14.63 (14.18, 15.09)* | *1 (ref.)* |  | *1 (ref.)* |  |
|  | *Overall off* | *94,622* | *4,930* | *52.10 (50.66, 53.57)* | *3.56 (3.20, 3.97)* | *<0·001* | *3.87 (3.51, 4.28)* | *<0·001* |
|  |  |  |  |  |  |  |  |  |
| Model 2 | Weeks 1-4 on | 15,171 | 355 | 23.41 | 1.85 (1.26, 2.42) | <0.001 | 1.83 (1.38, 2.43) | <0.001 |
|  | Rest on (ref.) | 252,858 | 3,389 | 13.40 | 1 (ref.) |  | 1 (ref.) |  |
|  | Week 1-4 off | 14,892 | 2,119 | 142.28 | 10.62 (9.04, 12.47) | <0.001 | 11.07 (9.62, 12.74) | <0.001 |
|  | Rest off | 82,297 | 3,036 | 36.89 | 2.75 (2.39, 3.18) | <0.001 | 3.11 (2.74, 3.52) | <0.001 |

**^a^** For models 1 and 2, the average from 50 imputed datasets is presented.

**^b^** For models 1 and 2, IRRs are adjusted for sex, age group (time-varying), health board, deprivation quintile, cumulative number of previous non-fatal overdoses (time-varying), and type (time-varying).

McAuley, Fraser R, Glancy M, Yeung A, Jones HE, Vickerman P, et al. Mortality among individuals prescribed opioid-agonist therapy in Scotland, UK, 2011–20: a national retrospective cohort study. Lancet Public Health. 2023 Jul 1;8(7):e484–93. doi:10.1016/S2468-2667(23)00082-8 PubMed PMID: 37295452.

Glancy M, McAuley A, Palmateer N, Trayner K, Yeung A, Barnsdale L, et al. Risk of drug-related mortality among people receiving opioid-agonist treatment during the COVID-19 pandemic in Scotland. Int J Drug Policy. 2025 Sep 1;143:104912. doi:10.1016/j.drugpo.2025.104912
